# MRI osteitis predicts cartilage damage at the wrist in RA: a three-year prospective 3T MRI study examining cartilage damage

**DOI:** 10.1186/ar4462

**Published:** 2014-01-30

**Authors:** Fiona M McQueen, Alexandra McHaffie, Andrew Clarke, Arier C Lee, Quentin Reeves, Barbara Curteis, Nicola Dalbeth

**Affiliations:** 1 Department of Molecular Medicine and Pathology, Faculty of Medical and Health Sciences, University of Auckland, 85 Park Rd, Grafton, Auckland, New Zealand; 2 Department of Radiology, Auckland City Hospital, Auckland District Health Board, Auckland, New Zealand; 3 Department of Epidemiology and Biostatistics, University of Auckland, Auckland, New Zealand; 4 Bone and Joint Research Group, Department of Medicine, University of Auckland, Auckland, New Zealand; 5 Department of Rheumatology, Greenlane Clinical Centre, Auckland District Health board, Auckland, New Zealand

## Abstract

**Introduction:**

Cartilage damage impacts on patient disability in rheumatoid arthritis (RA). The aims of this magnetic resonance imaging (MRI) study were to investigate cartilage damage over three years and determine predictive factors.

**Methods:**

A total of 38 RA patients and 22 controls were enrolled at t = 0 (2009). After 3 years, clinical and MRI data were available in 28 patients and 15 controls. 3T MRI scans were scored for cartilage damage, bone erosion, synovitis and osteitis. A model was developed to predict cartilage damage from baseline parameters.

**Results:**

Inter-reader reliability for the Auckland MRI cartilage score (AMRICS) was high for status scores; intraclass correlation coefficient (ICC), 0.90 (0.81 to 0.95) and moderate for change scores (ICC 0.58 (0.24 to 0.77)). AMRICS scores correlated with the Outcome MEasures in Rheumatoid Arthritis Clinical Trials (OMERACT) MRI joint space narrowing (jsn) and X-Ray (XR) jsn scores (r =0.96, P < 0.0001 and 0.80, P < 0.0001, respectively). AMRICS change scores were greater for RA patients than controls (P = 0.06 and P = 0.04 for the two readers). Using linear regression, baseline MRI cartilage, synovitis and osteitis scores predicted the three-year AMRICS (R^2^ = 0.67, 0.37 and 0.39, respectively). A multiple linear regression model predicted the three-year AMRICS (R^2^ = 0.78). Baseline radial osteitis predicted increased cartilage scores at the radiolunate and radioscaphoid joints, P = 0.0001 and 0.0012, respectively and synovitis at radioulnar, radiocarpal and intercarpal-carpometacarpal joints also influenced three-year cartilage scores (P-values of 0.001, 0.04 and 0.01, respectively).

**Conclusions:**

MRI cartilage damage progression is preceded by osteitis and synovitis but is most influenced by pre-existing cartilage damage suggesting primacy of the cartilage damage pathway in certain patients.

## Introduction

The progression of structural damage in rheumatoid arthritis (RA) is of great importance to rheumatologists and patients alike as it is associated with the development of joint deformity and eventually with disability [1]. Recent work has suggested that cartilage damage, as reflected by joint space narrowing (jsn), is more closely associated with irreversible physical disability than bony erosion [2] and is, therefore, a worthy focus of study, both to explore its use as an outcome measure in clinical trials and to improve our understanding of the pathological processes that drive rheumatoid joint damage. The Sharp van der Heijde score quantifies damage using two components, namely bone erosion and jsn, and is the most commonly used outcome measure in clinical trials [3]. Increasingly, magnetic resonance imaging (MRI) scanning is taking the place of X-ray (XR) for the assessment of RA as it is a more sensitive instrument for detecting bone erosion and has the added advantage of being able to image inflammatory change within bone (osteitis) and synovium (synovitis), which are the precursors of joint damage [4]. In recent years, the imaging of cartilage using high field MRI scanning and dedicated cartilage-sensitive sequences has become feasible, not only at large joints, such as the knee [5], but at the informative small joints of the wrist where progressive cartilage loss can result in carpal collapse [6].

The Outcome Measures in Rheumatoid Arthritis Clinical Trials (OMERACT) rheumatoid arthritis magnetic resonance imaging score (RAMRIS) system [7] has been devised to quantify inflammation and damage in rheumatoid joints and is now being used in RA clinical trials [8,9]. When the RAMRIS was initially developed, MRI systems were not sophisticated and quantification of the thin cartilage layer overlying the carpal bones was found to be too unreliable for inclusion in the score [10]. More recently, we devised the Auckland MRI cartilage score (AMRICS) using 3T MRI technology and found this to provide reliable cartilage quantification [11] while the OMERACT group subsequently developed a similar and also reproducible MRI jsn score [12]. Few studies to date have examined cartilage change over time in rheumatoid wrists, although Peterfy *et al*. have recently reported on the Impact of Rituximab on Magnetic Resonance Imaging Evidence of Synovitis and Bone Lesions in Patients With Moderate or Severe Rheumatoid Arthritis (IMPRESS) trial where patients received rituximab and/or methotrexate and were scanned at zero, three and six months. They found that the OMERACT MRI jsn score increased over this period, mirroring the progression of erosive damage [8].

We have previously described a strong association between the finding of osteitis (MRI bone oedema) in RA patients at first presentation and subsequent radiographic jsn and bone erosion six years later [13]. In light of these data, we and others have suggested that damage to bone and cartilage may proceed from a bone-centred inflammatory focus rather than according to the accepted paradigm which implicates the inflamed synovium as the origin of pathology [14,15]. In the current study, our aims have been to use high field MRI scanning to extend our understanding of the processes generating cartilage damage in RA. Using AMRICS, we have examined cartilage damage progression over three years in RA patients compared with controls. We have then investigated for baseline clinical and MRI factors associated with damage progression using a modelling approach.

## Methods

### Patients and clinical assessments

Patients and controls were recruited with the approval of the New Zealand Multiregion Ethics Committee and all provided written informed consent. A total of 38 RA patients, including 22 with early disease (onset within two years or less), 16 with established RA and 22 healthy controls, were enrolled at t = 0 (2009). After three years, clinical and MRI data were available in 28 RA patients (15 with early and 13 with established RA) and 15 controls. RA patients were treated with standard therapies including non-steroid anti-inflammatory drugs (NSAIDs), conventional disease modifying anti-rheumatic drugs (cDMARDs) and biological disease modifying anti-rheumatic drugs (bDMARDs). At t = 3 years, disease activity was low in the “early” RA group (median disease duration 4.5 years and Disease Activity Score 28 (DAS28) of 3.07), moderate in the “established” RA group (median disease duration 20 years and DAS28 of 3.56), and when the 2 RA groups were combined the DAS28 was 3.42. Table 1 summarises patient demographics, medications and disease activity.

**Table 1:** Demographics, medications and disease activity for RA patients and controls

**Clinical features**	**Early RA**	**Late RA**	**Healthy controls**
	**(n = 15)**	**(n = 13)**	**(n = 15)**
Age, yrs (median, range)	57 (36 to 87)	69 (43 to 84)	51 (37 to 62)
Female: male	11:4	8:5	13:2
Duration of RA, months	54 (48 to 72)	240 (83 to 456)	
Ethnicity: Caucasian (%)	87	85	87
Anti-CCP antibody + VE (%)	93	92	
RF positive (%)	60	69	
**Medications – No. (%)**			
NSAIDs	4 (27)	3 (23)	
MTX alone*	3	1	
cDMARD combinations**			
MTX, SSZ, HCQ	0	2	
MTX, HCQ, LEF	1	0	
MTX, LEF	2	0	
MTX, LEF, HCQ	0	1	
MTX, LEF, SSZ	1	0	
MTX, SSZ	0	2	
MTX, HCQ	3	1	
MTX, IM gold, LEF	0	1	
HCQ, LEF	1	0	
Prednisone 2.5 to 10 mg/d	4 (27)	2 (15)	
bDMARDs***	1 (7)	4 (31)	
ETC	1	1	
ADA	0	3	
**Disease activity (median, range)**			
Tender joint count (68)	8 (1 to 36)	17 (0 to 40)	
Swollen joint count (66)	1 (0 to 6)	3 (0 to 8)	
Pain VAS (mm)	14 (2 to 91)	20 (0 to 47)	
Global VAS (mm)	2 (0 to 4)	2 (0 to 3)	
HAQ score	0.75 (0 to 1.75)	0.88 (0 to 2.5)	
PF SF - 36	65 (5 to 100)	65 (5 to 95)	
CRP (mg/l)	2 (1 to 36)	4 (1 to 13)	1.2 (1 to 9)
DAS28_CRP3v_	3.1 (1.5 to 4.8)	3.6 (1.9 to 4.8)	

ADA, adalimumab 40 mg fortnightly; anti-CCP, antibodies to cyclic citrullinated peptide; bDMARDs, biological disease modifying antirheumatic drugs; cDMARDs, conventional disease modifying anti-rheumatic drugs; CRP, C-reactive protein; DAS28CRP3v, disease activity score 28, 3 variable CRP; HAQ, health assessment questionnaire; HCQ, hydroxychloroquine 200 to 400 mg/day; IM gold, myocrisin 50 mg/month; LEF, leflunomide 10 to 20 mg/day; NSAIDs, nonsteroidal anti-inflammatory drugs; PF SF-36, physical function component of short form-36 score; RF, rheumatoid factor; VAS, visual analogue scale.

*MTX, methotrexate 10 to 25 mg/week; **SSZ, sulphasalazine 1 to 3 g/day; ***ETC, etanercept 50 mg/week.

### MRI scans

3T MRI scans of the dominant wrist were scored for MRI parameters of disease activity and joint damage, including synovitis, osteitis, bone erosion and cartilage loss in RA patients and controls at baseline and after three years. MRI images were obtained on a 3T scanner Philips MR Systems Achieve 3T, Koninklijke Philips Electronics NV, Eindhoven, The Netherlands. An eight-element Philips SENSE 3.0T Wrist Coil 8 Channel (Invivo Corp) Gainsville, Florida (receive only) was used. The dominant hand was placed in the wrist coil where it fitted snugly by the patient’s side, palm facing the body, thumb anteriorly. The field of view was restricted to the carpus, including the distal radioulnar joint (dRUJ), extending to the metacarpal (MC) bases but excluding the metacarpophalangeal (MCP) joints. MRI scans were performed using standard sequences appropriate for imaging cartilage specifically as already described [11]. Briefly, these included the following turbo spin echo sequences: T1 weighted (T1w) and T2 weighted (T2w) sequences with fat saturation (FS) using spectral adiabatic inversion recovery (SPAIR) in the axial and coronal planes and proton density (PD) coronals (without FS) including an ultra-high resolution sequence. T1wFS axial and coronal sequences were obtained post-intravenous gadolinium diethylenetriamine pentaacetic acid (GdDTPA) given at a standard dosage of 10 ml Omniscan (Gadodiamide; 5.0 mmol/10 ml or 2.87 g/10 ml; GE Healthcare, Inc., Princeton, NJ, USA). In controls, scans were performed without contrast for ethical reasons and no assessment of MRI synovitis was attempted.

### Scoring MRI scans for cartilage damage

Scoring of MRI scans was performed separately (blinded) by the same two radiologists (AM and AC) who had scored the original scans from t = 0, but without reference to these scans, using a previously validated scoring system with cartilage sensitive sequences [11]. The AMRICS was developed in 2010 and scores 0 to 4 for joint space narrowing at each of eight joints within the carpus, including the distal radio-ulnar, radiolunate, radioscaphoid, triquetrum-hamate, capitate-lunate joint, scaphotrapezoid joint, second metacarpal base-trapezoid joint and third metacarpal base capitate joints, giving a maximum possible total score of 32. The system follows that developed by van der Heijde *et al*. for radiographic joint space narrowing as follows: 0 (normal thickness); 1 (asymmetrical or minimal narrowing to maximum of 25%); 2 (definite narrowing with loss of up to 50% of the normal space); 3 (definite narrowing with loss of 50 to 99% of the normal space or subluxation) and 4 (absence of joint space, presumed ankylosis or complete luxation).

### Plain radiography

Plain radiographs of the hands and feet were obtained in 28 RA patients and of the hands alone in 12 normal controls. Radiographs were scored as pairs (known chronological order) by a rheumatologist (ND, Reader 3), blinded to clinical and MRI data, for erosions and jsn using the Sharp van der Heijde (SvdH) score [3].

### Statistical analysis

Inter-reader reliability for MRI cartilage change scores (Auckland score) [11] was determined using absolute agreement intraclass correlation coefficients (ICCs) for a two-way random effects model. In addition, scans were scored using the OMERACT jsn MRI score [12] and correlations between the two scoring systems were examined using Pearson’s correlation coefficient. Subsequent analyses used data from Reader 1 (AM). T-test was used to compare the change in cartilage score between RA and controls. Pearson’s correlation coefficients were sought between MRI cartilage scores and radiographic jsn and total SvdH scores. Clinical and MRI parameters were examined to devise a model to predict cartilage damage after three years using a multiple linear regression method. The influence of therapy was also examined in the model including effects of methotrexate, methotrexate/sulphasalazine/hydroxychloroquine (triple therapy), anti-tumour necrosis factor (anti-TNF) therapy and prednisone. Effects of anti-CCP and rheumatoid factor positivity were also examined in the model. Simple linear regression was used to determine whether individual sites of cartilage loss were associated with preceding inflammation in adjacent bones seen as MRI bone oedema (osteitis) or joints (synovitis). The effects of anti-CCP and RF status were also examined in this analysis. Two-sided *P*-values less than 0.05 were used to determine statistical significance and all confidence intervals were given at a two-sided 95% level. All analyses were conducted using SAS for Windows version 9.3. Foundation for Microsoft® Windows® Copyright © 2012, SAS Institute Inc., Cary, NC, USA.

## Results

### Reliability of MRI cartilage scores and correlation with OMERACT MRI jsn scores

**Figure 1:**
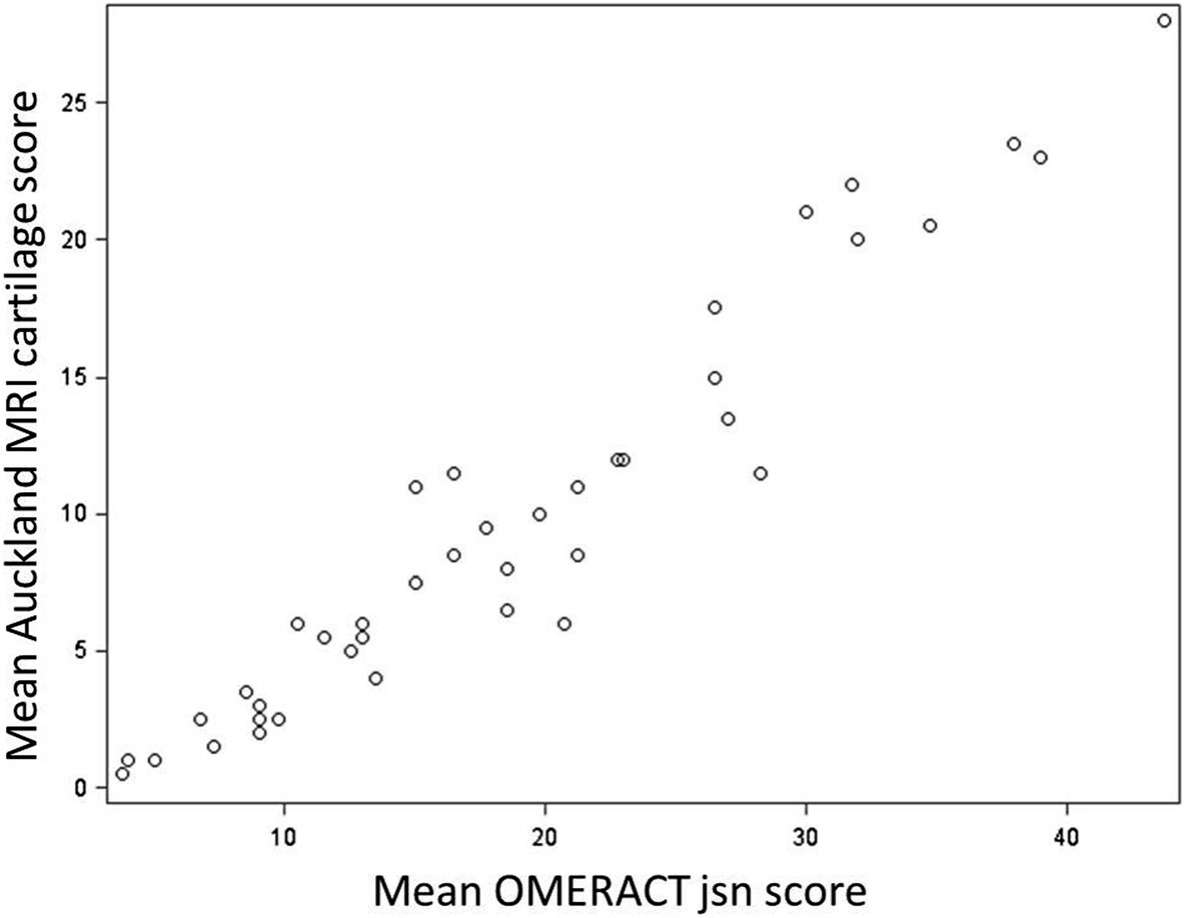
Auckland MRI cartilage score is highly correlated with OMERACT MRI jsn score (r = 0.96).

Inter-reader reliability for cartilage scores was high with an ICC of 0.9 (95% CI: 0.81 to 0.95). Inter-reader reliability for cartilage change scores was moderate (ICC 0.58 (95% CI: 0.24 to 0.77)) using the difference between 2012 cartilage scores and previous baseline cartilage scores in the database (without re-reading scans in a paired fashion). Intra-reader reliability was assessed at baseline and was 0.98 (95% CI: 0.96 to 1.00) for Reader 1 and 0.94 (95% CI: 0.87 to 1.00) for Reader 2 [11]. At three years (2012), Auckland MRI cartilage scores for both readers were highly correlated with OMERACT cartilage scores (r = 0.94 for each). When the mean Auckland MRI cartilage score was compared with the mean OMERACT MRI jsn score the correlation was even stronger (r = 0.96, *P* < .0001). Figure 1 shows this diagrammatically.

### Change in MRI cartilage scores and XR jsn over time

**Figure 2:**
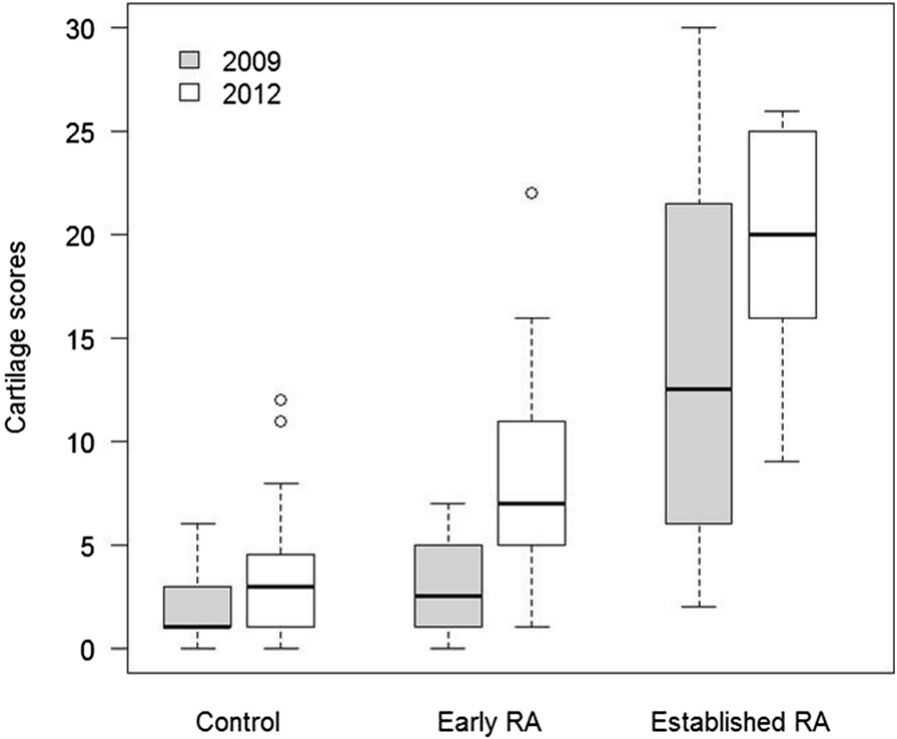
Progression of cartilage scores over three years in controls and RA patients (Reader 1 data).

MRI cartilage scores at t = 0 (2009) and t = 3 years (2012) for all three groups are shown in Figure 2 (Reader 1 data). When early and late RA groups were combined (N = 28), there was a significant change in MRI cartilage score over this period (mean change = 5 U, SE 0.93, Pr > |t| <0.0001). MRI cartilage change scores were then analysed separately for both readers and results are shown in Table 2. For Reader 1 data, there was some evidence suggesting a greater increase in cartilage scores in the RA group compared with controls (*P* = 0.067) and for Reader 2 data, the difference reached significance (*P* = 0.038). Changes in XR jsn scores in patients and controls (Reader 3) are also shown in Table 2 and these were significantly different (*P* = 0.04). At t = 3 years MRI cartilage scores were highly correlated with XR jsn scores (r = 0.80, *P* <0.0001).

**Table 2:** MRI cartilage score and in XR jsn over three years: RA vs controls

		Δ**AMRICS Reader 1**				
	**N**	**Mean**	**SE**	**SD**	**SRM**	Pr > |t|*
RA pts	28	5.00	0.93	4.92	1.02	0.0672
Controls	15	2.33	0.85	3.29	0.71	
		Δ**AMRICS Reader 2**				
RA pts	28	4.21	0.79	4.17	1.01	0.038
Controls	15	1.73	0.56	2.19	0.79	
		Δ**XR jsn Reader 3**				
RA pts	27	4.63	1.43	7.44	0.62	0.042
Controls	12	0.08	0.08	0.29	0.29	

ΔAMRICS, change in Auckland MRI cartilage score from baseline (2009) to three years (2012); ΔXR jsn, change in X-Ray joint space narrowing (Sharp van der Heijde score, hands only) from baseline to three years; SD, standard deviation; SE, standard error; SRM, standardised response mean.

*P-value for significance of difference between RA patients and controls.

### Baseline predictors of three-year cartilage and erosion scores: simple linear regression analysis

**Figure 3:**
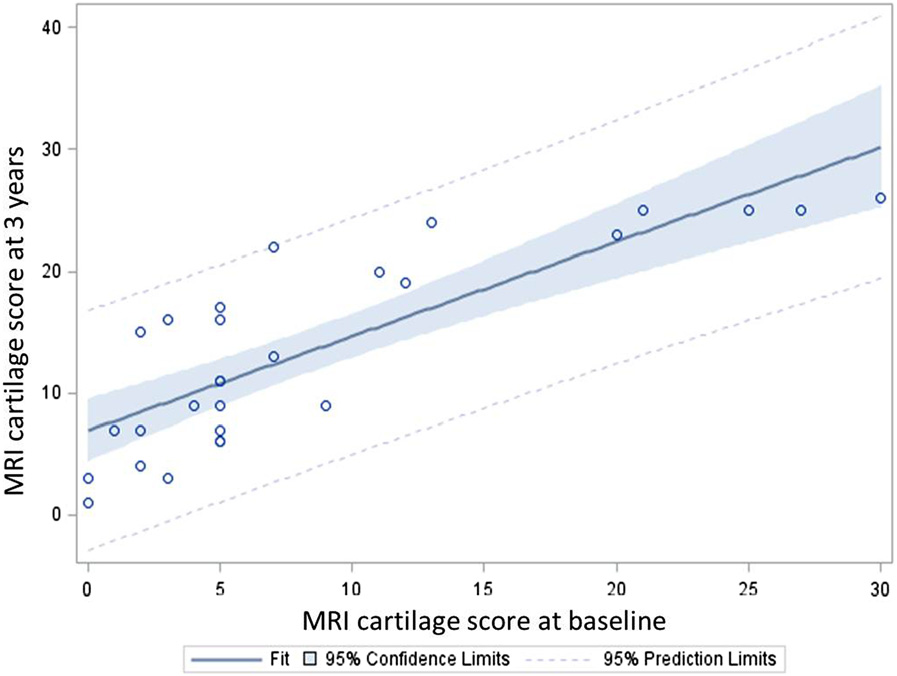
**Fit plot for Reader 1: baseline MRI cartilage scores predicted three-year cartilage scores (*P *** **<0.0001).**

We investigated whether the change in cartilage scores over time for the RA group was associated with baseline variables including clinical and MRI measures of disease activity and damage, using simple and multiple linear regression analyses. Results for simple linear regression are shown in Table 3. The baseline MRI cartilage score was the most significant determinant of the three-year cartilage score with strong positive relationship, (*P* <0.0001) and this is graphically represented in Figure 3. The baseline MRI erosion score was the most significant determinant of the three-year erosion score (R^2^ = 0.87) and also with strong positive relationship. All baseline (2009) MRI scores were strongly correlated with each other, as were three-year (2012) MRI scores and baseline score (Additional file 1: Table S1).

**Table 3:** Simple linear regression analysis to determine influence of baseline factors on damage outcomes (RA patients)

**Outcome = three-year MRI cartilage score**					
**Parameter**	**Estimate**	**SE**	**t**	**Pr > |t|**	**R2**
RA duration (years)	0.04	0.01	4.38	0.0002	0.42
Sex F (ref = M)	−1.67	3.26	−0.51	0.61	0.01
Age	0.13	0.11	1.22	0.23	0.05
DAS28CRP	2.39	1.52	1.57	0.1282	0.09
Anti-CCP positive	0.00	0.02	0.25	0.8056	0.00
HAQ	3.06	2.28	1.34	0.1913	0.06
MRI cartilage score	0.77	0.11	7.32	<.0001	0.67
MRI erosion score	0.52	0.11	4.77	<.0001	0.47
MRI bone oedema score	0.67	0.16	4.11	0.0004	0.39
MRI synovitis score	3.04	0.78	3.90	0.0006	0.37
**Outcome = 3 year MRI erosion score**					
RA duration (years)	0.06	0.01	5.62	<.0001	0.55
Sex F (ref = M)	-2.89	4.32	-0.67	0.50	0.02
Age	0.35	0.13	2.71	0.01	0.22
DAS28CRP	0.21	2.11	0.10	0.92	0.00
Anti-CCP positive	-0.03	0.02	-1.41	0.17	0.07
HAQ	1.96	3.11	0.63	0.53	0.02
MRI cartilage score	0.88	0.18	4.98	<.0001	0.49
MRI erosion score	0.95	0.07	13.15	<.0001	0.87
MRI bone oedema score	1.20	0.15	7.98	<.0001	0.71
MRI synovitis score	5.34	0.78	6.85	<.0001	0.64

DAS28, Disease Activity Score 28; CCP, cyclic citrullinated peptide; CRP, C-reactive protein; HAQ, health assessment questionnaire; RA, rheumatoid arthritis.

### A model to predict the three-year cartilage score; multiple linear regression analysis

A model was devised using multiple linear regression analysis, including clinical and MRI measures of disease activity, to determine baseline factors associated with the MRI cartilage score after three years. Table 4 shows the optimal model which included the following baseline parameters: disease duration, age, sex, DAS28CRP, RF positivity, HAQ score and MRI cartilage score. The R^2^ of the total model was 0.78, indicating that 78% of the variance observed in the MRI cartilage score at three years was explained by the model. The effect of substituting anticitrullinated peptide antibody (ACPA) status for RF was to slightly reduce the predictive power to 0.76. Without ACPA or RF in the model, the R2 was 0.75. Because all MRI scores at baseline were highly correlated, it was only appropriate to include one score in the model and the baseline cartilage score was chosen as this was the most influential factor on univariate analysis (R^2^ = 0.67). If the MRI bone oedema score was included instead, R^2^ for the total model was 0.65.

**Table 4:** Multiple linear regression model predicting the three-year MRI cartilage score*

**Baseline parameters**	**Estimate**	**SE**	**Pr > |t|**
Disease duration (years)	0.01	0.01	0.2916
Sex F (ref = M)	−3.10	2.08	0.1522
Age	0.02	0.07	0.7494
DAS28_CRP_	0.29	1.22	0.8127
RF	2.94	1.78	0.115
HAQ score	0.75	1.57	0.6379
MRI cartilage score	0.67	0.14	0.0001

*The R^2^ of the total model was 0.78, indicating 78% of the variance observed in the MRI cartilage score at three years was explained by the model. DAS28, Disease Activity Score 28; HAQ, health assessment questionnaire; RF, rheumatoid factor.

**Figure 4:**
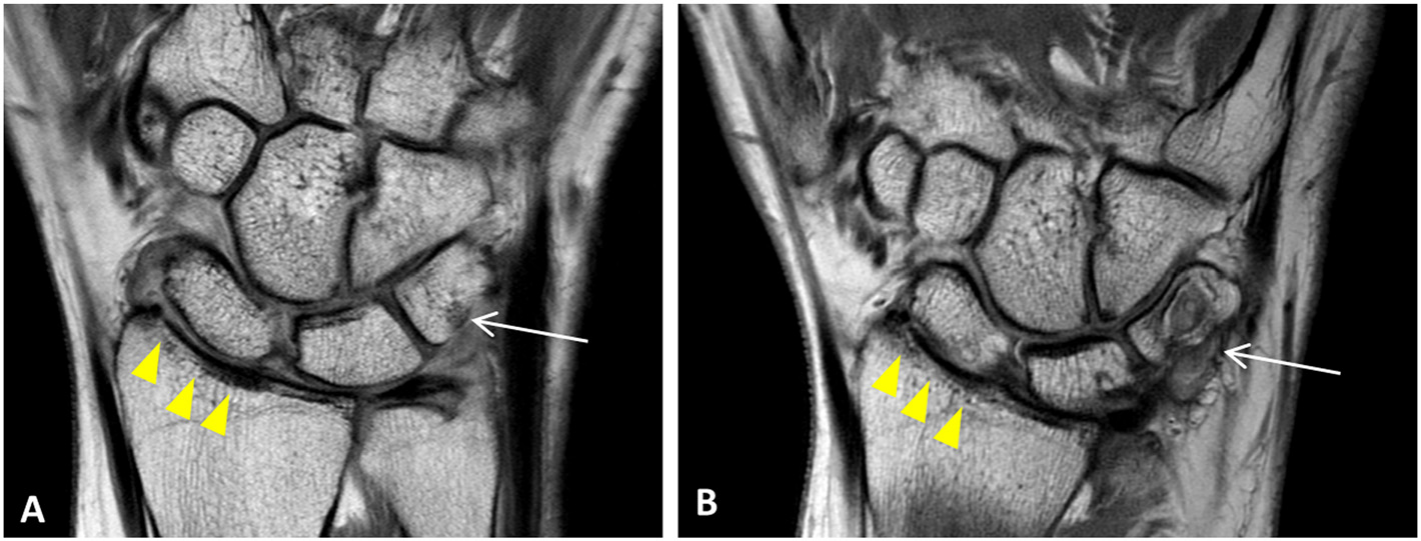
**Coronal proton density MRI wrist scans. A)** Baseline MRI scan (2009) shows cartilage thinning at the radiolunate (score = 2 ) and radioscaphoid joints (score = 1) (arrowheads) **B)** At 3 years (2012), the cartilage space narrowing has progressed; radiolunate score = 3 and radioscaphoid joint score = 3 (arrowheads). A new erosion (white arrow) is also now seen to involve the triquetrum at the site of bone oedema at baseline.

We then examined whether there was any effect from medication on the model in terms of predicting progression of cartilage damage. There was no significant effect from any of the medication combinations (including triple therapy, corticosteroid therapy and anti-TNF therapy) although there were only small numbers of patients in each group (N = 3, 6 and 5 patients, respectively, Table 1). When all methotrexate/cDMARD combinations were examined as a group, there was an effect on the model so that three-year cartilage scores were on average 5.1 U higher in these patients than in those not on this therapy. This is likely to represent confounding by indication (more active disease treated more intensively) as discussed below. Figure 4 shows progression of MRI cartilage damage in a patient maintained on methotrexate alone (intolerant to other DMARDs).

### Osteitis and synovitis predict cartilage score at neighbouring joints after three years

**Figure 5:**
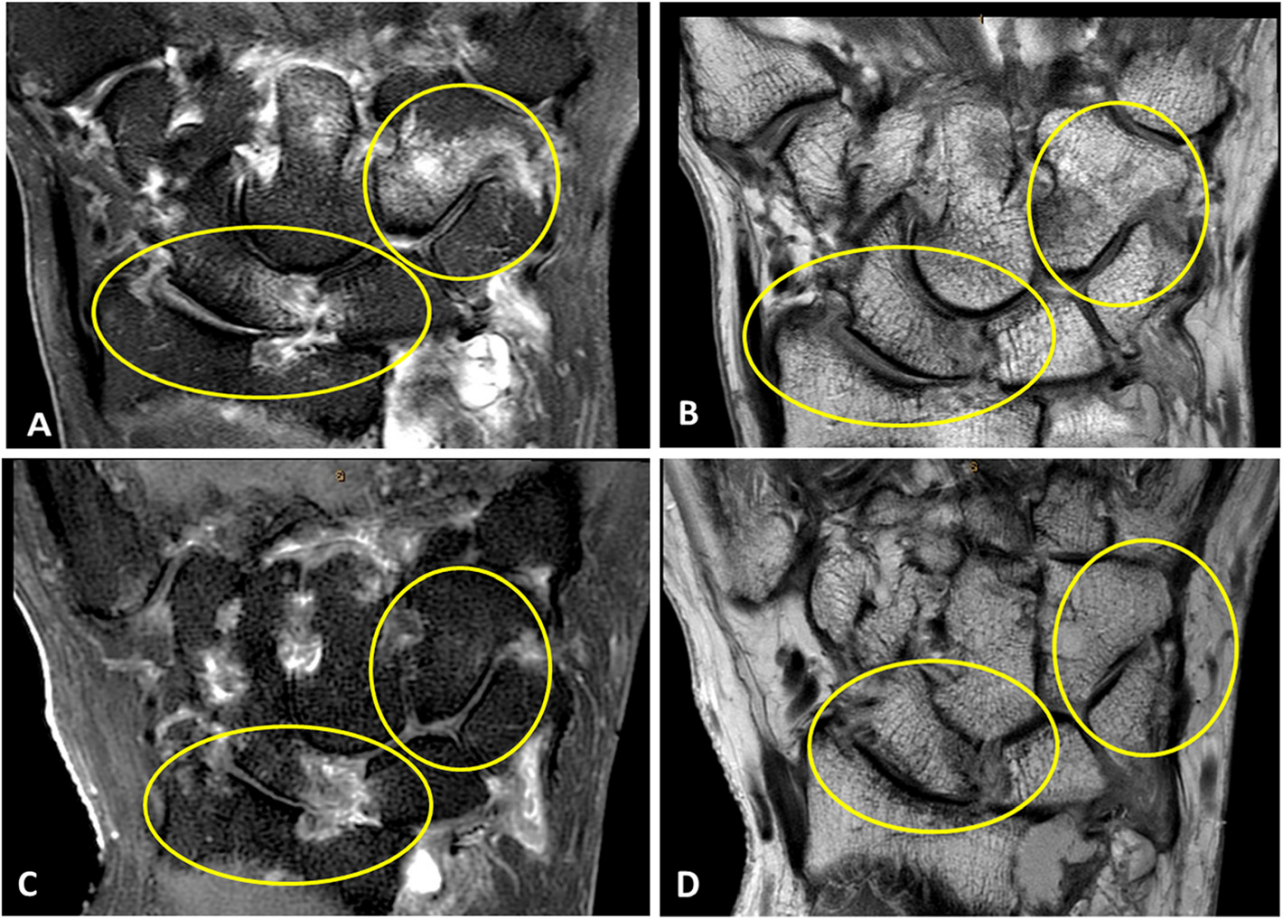
**Marked progression of cartilage thinning is associated with high levels of osteitis at baseline. A)** Coronal T2 FS image of the wrist at baseline (2009) showing bone oedema at the scaphoid and radius with an early erosion at the ulnar aspect of the joint (lower circle). Florid bone oedema is also seen involving the hamate (upper circle). **B)** Coronal PD ASY SENSE sequence showing asymmetrical reduction in radioscaphoid cartilage (lower circle) and normal cartilage space at the triquetrum-hamate joint (upper circle). **C)** and **D)** show equivalent sequences from the same patient after three years (2012) indicating that bone oedema has subsided but there has been marked progression in cartilage thinning at both radioscaphoid and triquetrum-hamate joints.

Bone oedema/osteitis at the radius and lunate (separately and summed) was investigated for associations with three-year cartilage scores at the radiolunate joint. Similarly, osteitis at the radius and scaphoid (separately and summed) was investigated for associations with three-year cartilage scores at the radioscaphoid joint. Data are presented in Table 5 and revealed a highly significant association for osteitis at the radius and the three-year radiolunate cartilage score (*P* = 0.0001). Similarly, osteitis at the radius at baseline was associated with the radioscaphoid cartilage score after three years (*P* = 0.0012). When the effects of ACPA or RF positivity were factored into this analysis, there was no significant effect (data available on request). Figure 5 shows progression in cartilage thinning at the radiolunate and radioscaphoid joints over the three-year period in a patient who had prominent osteitis at the radius scored at baseline. Similar to the findings for osteitis, we found evidence that baseline synovitis scores were predictive of cartilage scores at neighbouring joints, three years later. Synovitis scores at the three sites measured according to the RAMRIS system in 2009 (radioulnar joint, radiocarpal joint and intercarpal-carpometacarpal joints) were examined for an effect on the 2012 cartilage score at adjacent joints. Again there was an association for each joint region described above with *P*-values of 0.001, 0.04 and 0.01, respectively. We also explored prediction of cartilage change (ΔAMRICS) over three years from baseline parameters but found no association for synovitis or osteitis, either from sum scores (*P* = 0.52 and 0.63, respectively), or when individual sites were examined separately (*P* = NS), data not shown.

**Table 5:** Simple linear regression shows baseline bone oedema predicts three-year cartilage score

**Prediction of radiolunate cartilage score (2012)**			
Baseline bone oedema (2009)	Estimate	SE	Pr >|t|
Distal radius	1.40	0.31	0.0001
Lunate	0.19	0.30	0.518
Sum score for distal radius and lunate	0.45	0.19	0.0247
**Prediction of radioscaphoid cartilage score (2012)**			
Baseline bone oedema (2009)	Estimate	SE	Pr >|t|
Distal radius	1.10	0.30	0.0012
Scaphoid	0.84	0.27	0.0045
Sum score distal radius and scaphoid	0.55	0.15	0.001

### C-progressors and E-progressors

**Figure 6:**
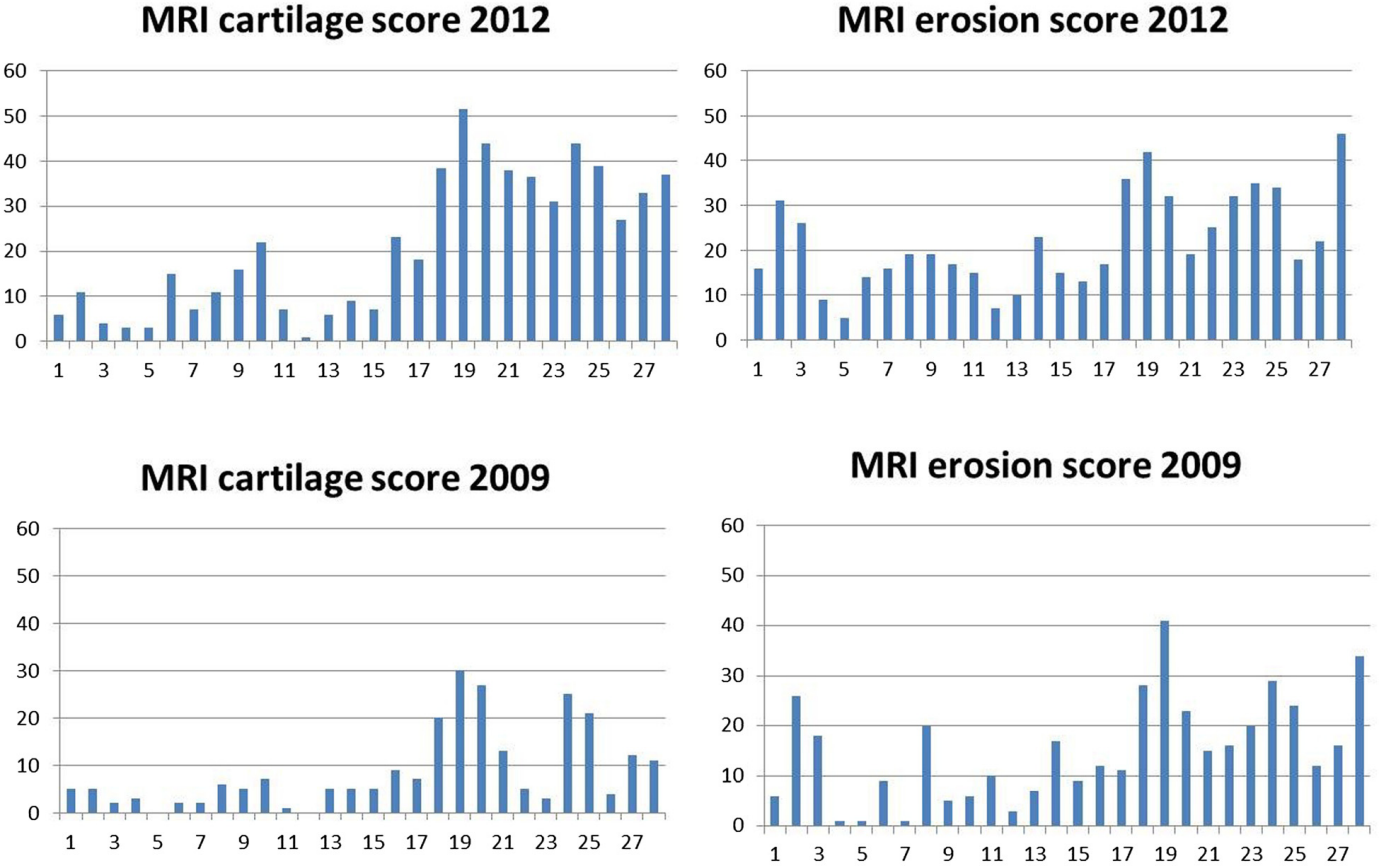
MRI cartilage and erosion scores at baseline and three years: each patient as a bar.

We examined our data to see whether there was any support for the notion that patients favour one particular damage pathway over the other, that is, that those who develop erosions tend to erode further (E-progressors) while those who damage cartilage continue preferentially in that manner (C-progressors). Using multiple linear regression with an outcome of cartilage damage at three years as alluded to above, the strongest predictor for the cartilage score was the baseline cartilage score (R^2^ = 0.67). The baseline MRI erosion score was also predictive but to a lesser degree (R^2^ = 0.47). When the outcome of bone erosion score was used, the strongest predictor was the baseline erosion score (R^2^ = 0.87, *P* <0.0001), while the baseline cartilage score was not quite as strongly predictive (R^2^ = 0.49, *P* = 0.01). These data would support but do not prove the proposal above. Figure 6 shows progression of cartilage and erosion scores for each patient from baseline to three years.

## Discussion

This is the largest prospective MRI study of cartilage damage in RA to be conducted outside of a clinical trial setting, reflecting “real life” disease progression. The patients studied were recruited from rheumatology outpatient clinics and represent a typical spectrum of disease severity and duration, on a variety of medications, including predominantly combination traditional DMARD therapy. A relatively small number were taking bDMARDs, influenced by the New Zealand government funding system which only allows prescription of biological agents to patients with erosive RA who have failed multiple combinations of traditional DMARDs. The first aim of the study was to determine the operating characteristics of the AMRICS in a longitudinal setting. We found this scoring system performed well with high inter-reader reliability for three-year status scores although reliability was only moderate for cartilage change scores, probably influenced by the fact that the three-year images were read separately without reference to previous scans, which is a more stringent test of scoring accuracy than reading paired images in known chronological order [16]. The standardised response means were high indicating good responsiveness of the score for detecting cartilage change. This compares with the findings of Haavardsholm *et al*. investigating MRI and XR parameters for detecting structural change in rheumatoid joints, where change in the RAMRIS erosion score showed high sensitivity (SRM 0.89) as did the SvdH total score (SRM 0.94) [17].

We found very strong correlations between the AMRICS and the OMERACT MRI jsn score [12] for both readers, and between AMRICS and the XR SvdH jsn score [3], providing construct validity for the MRI cartilage score [18]. We felt it important to include healthy controls, some of whom may have osteoarthritis (OA) as a comparison group as some RA patients also have concomitant OA that could affect cartilage thickness and have the potential to progress over time. As we suspected, there was measurable cartilage damage progression in some controls. This did affect RA-relevant sites such as the radiolunate and radioscaphoid joints where cartilage thinning was observed in several individuals related to degenerative joint disease and also ulnar-lunate cartilage impaction, a not uncommon finding in asymptomatic wrists [19]. Interestingly, when RA and control groups were compared for cartilage damage progression, only one reader found a significant difference between them, while for the other reader there was a statistical trend only (*P* = 0.06). The XR jsn measure revealed very similar separation between patients and controls and just reached significance using a different reader. These data emphasise the importance of including controls in any study of cartilage damage progression in RA and provide some indication of minimum group size for future clinical trials. That MRI and XR produced similar results for assessing cartilage damage progression could be taken as an argument for using radiography alone as an outcome measure. However, Peterfy *et al*. noted that interposition of synovial tissue or joint effusion between articular surfaces can decrease the accuracy of XR jsn measurements implying that the direct measurement of cartilage thickness from MRI scans is advantageous [8]. A further advantage of using MRI in assessing RA damage is that it provides additional information about preceding osteitis and synovitis which may also impact upon management decisions.

The second major aim of this project was to explore baseline predictors of three-year MRI cartilage scores. We assessed measures of joint inflammation, including osteitis and synovitis, as well as damage, including bone erosion and cartilage thinning, individually and combined with clinical scores to create a best-fit model to explain this score. The best individual predictor was the baseline MRI cartilage score. When all available baseline data were used in a model, we were able to explain 76% of the variance observed in the three-year cartilage score but 67% of this was from the baseline cartilage score. These data indicate that those patients who developed the most severe cartilage damage after three years were those who already had significant damage at baseline. These findings are in agreement with those of van der Heijde *et al*. studying radiographic jsn in 870 joints from the ASPIRE trial [20]. They found that for joints with jsn present, there was a preference for worsening of jsn while for joints with evidence of erosive damage, there was a preference for continuing erosion. Thus, although both bone erosion and cartilage thinning contribute to rheumatoid joint damage, these could be separate processes that proceed via different pathological pathways [21]. The effect of the RANKL inhibitor denosumab in abrogating erosive progression without affecting cartilage damage progression is given as further evidence for this hypothesis [22].

The modelling outcome described above indicates that pre-existing cartilage damage can be used to predict a worsening of that damage. Clearly this reflects the cohort of patients studied who had established and sometimes very longstanding disease. If inflammatory variables were examined instead, baseline osteitis and synovitis were also independent predictors of cartilage damage at three years and on a site-by-site basis were highly likely to be associated with subsequent cartilage damage. This is consistent with an inflammation-driven process leading to cartilage damage. However, we were unable to predict the extent of progression of cartilage change using these variables as, for example, osteitis was very common adjacent to joints that progressed as well as those that did not progress. This may have been for one or more of the following reasons; the AMRICS had relatively low sensitivity for detecting cartilage change, joints were not examined over a sufficiently long period for damage to become apparent, or because other factors, such as medication use, might have influenced damage progression [23]. We did not factor in the effect of tenosynovitis on cartilage damage as this feature was not scored on baseline or three-year scans in our cohort. However, Navalho *et al*. [24] showed that MRI tenosynovitis at the hands and wrists was predictive of progression to criteria-positive RA in patients with very early disease, and Lillegraven *et al*. [25] found that ultrasound-detected tenosynovitis of extensor carpi ulnaris (ECU) was associated with progression in the RAMRIS erosion score at the distal ulna over one year. Taken together, these data suggest that a combination of synovitis, osteitis and tenosynovitis could be a useful “total inflammation score” to examine as a predictor of joint damage in future studies.

We did examine for an effect of medication use in the model, assuming that powerful anti-rheumatic agents might slow progression of cartilage damage. We found no effect from triple therapy, prednisone or anti-TNF agents, possibly because of small numbers in each group. When any combination of methotrexate-plus-cDMARDs was examined as a group, there was a significant effect on three-year cartilage scores, which were on average 5.1 U higher than for those not taking methotrexate/cDMARDs. This finding is in the opposite direction to what would be expected if we were observing an effect of therapy on cartilage damage. It is therefore likely that we are observing a “confounding by indication” effect as we reported in an earlier cohort of RA patients followed prospectively for the development of MRI erosions [26]. In that study, those on DMARDs (including methotrexate) were more likely to have erosive joint damage after one year, indicating that more aggressive medication use tends to be instituted in those with more active clinical disease.

## Conclusions

In summary, this study has answered some questions relating to cartilage damage in RA and raised others. We have confirmed that measuring progression of cartilage thinning over time is possible using MRI but our data suggest that plain radiography could be comparable in terms of separating patients from controls, when considering this endpoint alone. Longitudinal studies comparing MRI with XR and CT scanning, are needed to further investigate this issue. However, we have shown that MRI yields interesting information about inflammation preceding cartilage damage and the strong associations between baseline osteitis and synovitis and the three-year cartilage score suggest that these measures could be important imaging biomarkers to indicate those at highest risk for cartilage damage progression. One of the most interesting findings has been to support the notion that there are two damage pathways; one that leads to erosion of bone and one that leads to thinning of cartilage and that these seem to be favoured by individual patients (E-progressors and C-progressors, respectively). Further studies of the genetic and immunopathogenic characteristics of these two groups are warranted to improve our understanding of factors leading to joint damage in RA and to determine whether management strategies should be matched to the tissue damage target.

## Abbreviations

3-T MRI: 3 Tesla magnetic resonance imaging; ACPA: Anticitrullinated peptide antibody; AMRICS: Auckland MRI cartilage score; bDMARDs: biological disease modifying anti-rheumatic drugs; C-progressors: cartilage progressors; DAS28: Disease activity score (28 joints); dRUJ: distal radioulnar joint; E-progressors: Erosion progressors; FS: Fat saturation; GdDTPA: Gadolinium diethylenetriamine pentaacetic acid; HAQ: Health assessment questionnaire; ICC: Intraclass correlation coefficient; jsn: joint space narrowing; MC: Metacarpal; MCP: Metacarpophalangeal; ml: millilitre; mmol: millimole; NS: Not significant; OA: Osteoarthritis; OMERACT: Outcome Measures in Rheumatoid Arthritis Clinical Trials; PD: Proton density; RA: Rheumatoid arthritis; RAMRIS: Rheumatoid arthritis MRI score; RF: Rheumatoid factor; SE: standard error; SENSE: Sensitivity encoding; SPAIR: Spectral adiabatic inversion recovery; SRM: Standardised response mean; SvdH: Sharp van der Heijde; t: time; T1w: T1 weighted; T2w: T2 weighted; TNF: Tumour necrosis factor; U: units; XR: X-Ray; ΔAMRICS: change in Auckland MRI cartilage score.

## Competing interests

The authors declare that they have no competing interests.

## Authors’ contributions

FM conceived the idea for the study, developed the methodology, performed the analysis and wrote the manuscript. AMcH and AC read the MRI scans and contributed to developing the methods and review of the manuscript. AL helped formulate statistical methods, performed the analysis and reviewed the final paper. QR formulated MRI sequences and methods, reviewed scans and participated in writing the manuscript. BC was involved in patient recruitment and examination plus review of methodology and in writing the manuscript. ND read radiology, advised on methods and assisted in writing and reviewing the manuscript. All authors read and approved the final manuscript.

## Supplementary Material

Additional file 1: Table S1 Correlations between MRI scores at baseline and three years (Reader 1). Pearson’s correlation coefficients for association between baseline MRI scores (2009) and three-year MRI scores (2012) for Reader 1. Click here for file
