# Supplementary material for: MRI osteitis predicts cartilage damage at the wrist in RA: a three-year prospective 3T MRI study examining cartilage damage
Source: Arthritis Res Ther. 2014 Jan 30;16(1):R33. doi: 10.1186/ar4462 (PMC3978660; doi:10.1186/ar4462)
Supplement: Additional file 1: Table S1 — Correlations between MRI scores at baseline and three years (Reader 1). Pearson’s correlation coefficients for association between baseline MRI scores (2009) and three-year MRI scores (2012) for Reader 1. [file ar4462-S1.docx]

**Additional File 1**

**Table 1. on-line supplement**

Correlations between MRI scores at baseline and 3 years (Reader 1).

| **MRI scores** | **Statistical tests** | Cartilage 2009 | Cartilage 2009 | Bone oedema 2009 | Erosion 2009 | Synovitis 2009 |  |
| --- | --- | --- | --- | --- | --- | --- | --- |
| Cartilage 2012 | Correlation coefficient | 1 | 0.82 | 0.63 | 0.68 | 0.61 |  |
|  | *P* value |  | <.0001 | 0.0004 | <.0001 | 0.0006 |  |
|  | No. of observations | 28 | 28 | 28 | 28 | 28 |  |
| Cartilage 2009 | Correlation coefficient | 0.82 | 1 | 0.66 | 0.70 | 0.57 |  |
|  | *P* value | <.0001 |  | <.0001 | <.0001 | 0.0002 |  |
|  | No. of observations | 28 | 38 | 38 | 38 | 38 |  |
| Bone oedema 2009 | Correlation coefficient | 0.63 | 0.66 | 1 | 0.91 | 0.86 |  |
|  | *P* value | 0.0004 | <.0001 |  | <.0001 | <.0001 |  |
|  | No. of observations | 28 | 38 | 38 | 38 | 38 |  |
| Erosion 2009 | Correlation coefficient | 0.68 | 0.70 | 0.91145 | 1 | 0.87 |  |
|  | *P* value | <.0001 | <.0001 | <.0001 |  | <.0001 |  |
|  | No. of observations | 28 | 38 | 38 | 38 | 38 |  |
| Synovitis 2009 | Correlation coefficient | 0.61 | 0.57 | 0.86 | 0.87 | 1 |  |
|  | *P* value | 0.0006 | 0.0002 | <.0001 | <.0001 |  |  |
|  | No. of observations | 28 | 38 | 38 | 38 | 38 |  |
